# Associations between pre-pregnancy BMI, gestational weight gain, and prenatal diet quality in a national sample

**DOI:** 10.1371/journal.pone.0224034

**Published:** 2019-10-18

**Authors:** Haley W. Parker, Alison Tovar, Karen McCurdy, Maya Vadiveloo

**Affiliations:** 1 Department of Nutrition and Food Sciences, University of Rhode Island, Kingston, Rhode Island, United States of America; 2 Department of Human Development and Family Studies, University of Rhode Island, Kingston, Rhode Island, United States of America; Monash University, AUSTRALIA

## Abstract

This secondary analysis explored the association between gestational weight gain, pre-pregnancy body mass index (BMI), and prenatal diet quality in a United States national sample. The sample comprised 1322 pregnant women in the longitudinal Infant Feeding Practices Study II with Diet History Questionnaire data. Diet quality in the third trimester was assessed using the Alternative Healthy Eating Index for Pregnancy. Self-reported pre-pregnancy BMI (categorized as underweight<18.5, normal weight 18.5–24.9, overweight 25.0–29.9, and obese≥30.0) and total gestational weight gain were used to categorize adherence to the Institute of Medicine’s recommendations as inadequate, adequate, or excessive weight gain. Diet quality in pre-pregnancy BMI and gestational weight gain groups were compared using Tukey-adjusted generalized linear models adjusted for sociodemographic factors, Women, Infants, and Children participation, parity, and energy intake. Due to missing gestational weight gain data, sensitivity analyses with multiply imputed data were conducted. Women were on average 28.9 years old and of higher socioeconomic status (40% college graduates) and mostly non-Hispanic White (84%), and the mean Alternative Healthy Eating Index for Pregnancy score was 61.2 (of 130). Both pre-pregnancy BMI and gestational weight gain were inversely associated with diet quality scores (p<0.01). The interaction between pre-pregnancy BMI and gestational weight gain was significant (p = 0.04), therefore gestational weight gain models were stratified by BMI group. In stratified adjusted models, gestational weight gain was differently associated with diet quality scores (p<0.05) among women with underweight, normal weight, overweight, and obesity. The relationship between gestational weight gain and prenatal diet quality depended on pre-pregnancy BMI. For example, within women with normal weight, higher diet quality was observed in the adequate gestational weight gain group. Interventions to broadly improve prenatal diet quality are needed, however, resources can be used to target women with higher pre-pregnancy BMIs and women with inadequate or excessive gestational weight gain.

## Introduction

Considerable recognition has been given to the relationship between early life (i.e. from conception to age two) exposures and long-term health outcomes. Nutritional exposures during pregnancy (i.e. prenatal diet) influence the mother’s health as well as pregnancy outcomes [1–3], and the child’s growth trajectories and chronic disease risk [4–6]. Due to increased physiological adaptability during development, prenatal diet can influence the child’s long term health outcomes through numerous pathways [7]. For example, maternal overnutrition (e.g., exceeding energy needs) can result in altered insulin, glucose, and lipid metabolism in the infant [8–10], inadequate micronutrient intake can increase risk of low birthweight and pre-eclampsia [11,12], intake of oily fish may help reduce the child’s risk of developing allergies and asthma [13], and higher intake of highly processed foods has been associated with increased infant adiposity [14].

As there is greater recognition of the complexity of prenatal dietary exposures on fetal development, programing and subsequent long-term health outcomes, more research is needed to understand the determinants of prenatal diet quality (i.e. adherence to dietary recommendations). Currently, prenatal diet quality in the United States is low; pregnant women in the National Health and Nutrition Examination and Survey 2003–2012 scored 50.7 points out of 100 on the Healthy Eating Index-2010 [15], which is 8% lower than the average adult living in the United States [16] and about 30% lower than the ideal score for disease prevention [17]. At present, maternal overnutrition measured with pre-pregnancy body mass index (BMI) is a regularly screened risk factor due to consistent evidence of increased risk of adverse pregnancy and health outcomes in women who enter pregnancy with higher BMI’s [8,9,18]. In addition to increased risk of adverse outcomes, research indicates that women who begin pregnancy at higher BMI’s are also disproportionately impacted by poor prenatal diet quality [15,19,20], in a national sample, diet quality scores for women with pre-pregnancy obesity were approximately 6% lower than women with pre-pregnancy normal weight [15]. Yet while pre-pregnancy BMI is an important predictor of prenatal diet quality [15,19,20] and health outcomes [8,9,18], the measure of pre-pregnancy BMI as an indication of dietary risk assumes that diet will be static throughout the course of pregnancy, which is not supported by existing research [21,22]. Conversely, pregnancy reflects a time where dietary and lifestyle modifications are common [21–23], yet little research has explored whether gestational weight gain (GWG), a dynamic, pregnancy-related measure, is differently associated with prenatal diet quality than the static measure of pre-pregnancy BMI. Though a variety of lifestyle factors influence GWG trajectories, if both pre-pregnancy BMI and GWG are associated with prenatal diet quality, then both measures should be explored to examine the associations between maternal factors and health outcomes.

Although pre-pregnancy BMI is a static risk factor throughout pregnancy, public health efforts and epidemiological research have largely focused on this measure[24] due to increasing obesity prevalence over the past several decades [25]. While substantial research has focused on preventing excessive GWG, the role of prenatal diet as a determinant of GWG has been examined infrequently[26], despite its association with a variety of pregnancy and birth outcomes [27]. Given that trajectories of GWG during pregnancy have been shown to be influential on pregnancy and child health outcomes [28,29], monitoring deviance from the recommended GWG trajectories across pre-pregnancy BMI categories may offer an opportunity for more effective dietary interventions. Therefore, this study aimed to examine the associations between both pre-pregnancy BMI and GWG with prenatal diet quality to help identify groups at increased risk for poor prenatal diet quality relevant for public health interventions. We hypothesized that women who adhered to pre-pregnancy BMI and GWG recommendations (i.e. women who entered pregnancy at a normal weight BMI and had adequate GWG [30]) would have higher prenatal diet quality.

## Methods

This secondary data analysis was completed using publicly available data from the Infant Feeding Practices Survey (IFPS) II, a longitudinal cohort study conducted by the Centers for Disease Control and Prevention and the Food and Drug Administration which aimed to collect data on infant feeding practices, maternal diet, sociodemographic factors, and healthcare [31]. Details regarding IFPS II methods have been previously reported [31]. Briefly, about 4,900 women in their third trimester of pregnancy were recruited in 2005 from a national consumer panel of >500,000 households. To be included in IFPS II, mothers and infants needed to be free of health conditions that impacted feeding, and infants needed to be born ≥34 weeks of gestation with a birthweight ≥5 lb (2268 g) from a singleton pregnancy and had not stayed in the intensive care unit longer than 3 days. Follow-up surveys were mailed approximately monthly during the infant’s first year, in addition to a 6-year follow-up survey. This analysis included self-reported data from the prenatal surveys sent during the third trimester (informing demographics, health information, diet history) and the neonatal survey sent immediately following birth (informing GWG). This secondary data analysis was deemed exempt from review by the Institutional Review Board at the University of Rhode Island. The original study was approved by the Food and Drug Administration's Research Involving Human Subjects Committee and the US Office of Management and Budget. All data was de-identified prior to public release.

### Exposure variables

Pre-pregnancy height and weight, used to calculate BMI, were reported in the prenatal survey; BMI values were categorized according to the Center for Disease Control and Prevention’s criteria (<18.5 is underweight, ≥18.5–24.9 is normal weight, ≥25.0–29.9 is overweight, and ≥30.0 is obese) [32]. Women were asked to report how much weight they gained during their pregnancy on the neonatal survey completed when the infant was approximately 1 month old. A categorical variable was created to reflect adherence to Institute of Medicine’s 2009 guidelines GWG ranges which are dependent on pre-pregnancy BMI [30]; women who gained within their designated pre-pregnancy BMI-dependent GWG range were categorized as adequate, weight gain below the range was categorized as inadequate, and above the range was categorized as excessive.

### Outcome variable

During the third trimester of pregnancy, a subsample (n = 1,444) of IFPS II participants were selected to complete the Diet History Questionnaire [33], a 149-question food frequency questionnaire reflecting the previous month of intake, modified for pregnancy to include foods (e.g. fish) and supplements of interest. The Diet History Questionnaires were analyzed using the Diet*Calc software, producing daily intake estimates of various nutrients and food groups [33]. Using data from responses to the Diet History Questionnaire and Diet*Calc output, prenatal diet quality was calculated using the Alternative Healthy Eating Index for Pregnancy (AHEI-P), a version of the Alternative Healthy Eating Index adapted for pregnancy by Poon et al [34].

The AHEI-P is comprised of 13 components, each worth 10 points and totaling a maximum score of 130 points. The components can be categorized as adequacy components, where increased intake is recommended, or moderation components, where restricted intake is recommended. The AHEI-P has 4 moderation (sugar sweetened beverages including fruit juice, red and processed meat, trans fat, and sodium) and 9 adequacy components (vegetables, whole fruit, whole grains, nuts and legumes, long chain fatty acids, polyunsaturated fatty acids, iron, folate, and calcium). Higher total and component scores reflect increased adherence to recommendations.

### Analyses

Prior to analyses, variable distributions were inspected for normality and cleaned of biologically implausible values. One extreme BMI observation (<10) was removed. A wide range of energy intakes,674–6264 kcals (2.8–29.2 MJ), were reported. Using a plausible prenatal energy intake range of 1075–4777 kcals (4.5–20 MJ) defined in a previous study [35], we eliminated observations outside of this range leaving an analytic sample of n = 1322 (original sample, n = 1444).

Age- and multivariable-adjusted (adjusted for age, energy intake, and other influential covariates) generalized linear models were used to compare prenatal diet quality scores among pre-pregnancy BMI and GWG groups. Covariates examined included education (high school or less, some college, college graduate), poverty income ratio (≤1.85, >1.85 and <4, ≥4), parity (primiparous or multiparous), age (continuous), race (non-Hispanic White, non-Hispanic Black, Other races), prenatal Women, Infants, and Children (WIC) participation status (yes or no), smoking status (current or non-smoker), and energy intake (in kcals/day), which were selected based on the literature [15,19,20,36]. Models were selected by singly adding covariates into the unadjusted model and examining both the covariate’s significance and influence on group mean scores. The multivariable-adjusted model for pre-pregnancy BMI included age, smoking, poverty income ratio, WIC participation, race, and energy intake as covariates and for the GWG model, age, poverty income ratio, smoking status, WIC participation, parity, and energy intake were included as covariates. In this complete case analysis, the analytic sample was limited to n = 908 women who reported GWG. Interactions were tested between variables with known associations including pre-pregnancy BMI and a) poverty income ratio [37] and b) energy intake and between GWG and a) pre-pregnancy BMI and b) race [30]; if significant, stratified analyses were conducted. When overall models were significant, Tukey-adjusted post-hoc testing was used to determine which groups were different. All analyses were completed in SAS 9.4 and p<0.05 was used to indicate significance.

### Sensitivity analyses

Approximately 31% (n = 414 out of 1322) of the analytic sample was missing GWG data. Descriptive statistics including means and frequencies were calculated and compared amongst women who reported GWG and women who did not report GWG using chi-square tests for categorical variables and t-tests for continuous variables. After eliminating participants who refused to report GWG on the neonatal survey (n = 49) from the analytic dataset, 10 datasets were multiply imputed (n = 1273) using a fully conditional specification regression model with PROC MI in SAS 9.4. The imputation model included the exposure variables (pre-pregnancy BMI and GWG), outcome variable (total diet quality score), and covariates for age, poverty income ratio, smoking status, WIC participation, parity, and energy intake. Sensitivity analyses were completed using the MIANALYZE procedure, where the imputed datasets were pooled and age- and multivariable-adjusted analyses were repeated on the imputed data.

## Results

The average age of the sample was 28.9±5.6 years and women were mostly college educated (40% with some college and 40% college graduates), Non-Hispanic White (84%), multiparious (71%), and non-smoking (90%) (**Table 1**). The average AHEI-P score was 61.2 out of 130 points, and most participants reported a pre-pregnancy BMI in the normal weight range (47%) followed by obese (25%), overweight (23%), then underweight (5%). For the 69% of participants who reported GWG, most did not meet the GWG guidelines with 46% exceeding them, 16% had inadequate GWG, and the remaining 38% had adequate GWG. Missingness of GWG data was significantly higher in women with lower education, from minority racial groups, participating in WIC, and smokers.

**Table 1:** Maternal characteristics for the subsample of 1,322 women in the Infant Feeding Practices Study II who completed the prenatal diet history questionnaire.

Maternal Characteristic	Mean ± standard deviation or n (%)			P-value for Chi-square or t-test
	Total (n = 1322)	GWG^a^ reporters (n = 908, 68.7%)	GWG non-reporters^b^ (n = 414, 31.3%)	
Age, y	28.9±5.6	29.1±5.3	28.6±6.1	0.1263
Education				0.0006
High school or less	250 (20.9%)	161 (13.5%)	89 (7.5%)	
Some college	472 (39.5%)	340 (28.5%)	132 (11.1%)	
College graduate	472 (39.5%)	367 (30.7%)	105 (8.8%)	
Race				0.0002
Non-Hispanic White	1071 (83.9%)	780 (61.1%)	291 (22.8%)	
Non-Hispanic Black	55 (4.3%)	27 (2.1%)	28 (2.2%)	
Other^c^	150 (11.8%)	97 (7.6%)	53 (4.2%)	
Multiparious	890 (70.9%)	648 (51.6%)	242 (19.3%)	0.0721
Poverty Income Ratio				0.0791
Low-income^d^	527 (41.0%)	359 (27.9%)	168 (13.1%)	
Middle-income^e^	524 (40.8%)	370 (28.8%)	154 (12.0%)	
High-income^f^	235 (18.3%)	179 (13.9%)	56 (4.4%)	
WIC^g^ Participation	366 (28.5%)	233 (18.2%)	133 (10.4%)	0.0005
AHEI-P total score^h^	61.2±10.8	61.3±10.7	61.2±11.0	0.9558
Nonsmokers	1149 (89.8%)	824 (64.4%)	325 (25.4%)	0.0066
Pre-pregnancy (BMI)^i^				0.5970
BMI <18.5	66 (5.2%)	44 (3.5%)	22 (1.7%)	
BMI ≥18.5–24.9	596 (47.0%)	431 (34.0%)	165 (13.0%)	
BMI ≥25.0–29.9	285 (22.5%)	209 (16.5%)	76 (6.0%)	
BMI ≥30.0	321 (25.3%)	224 (17.7%)	97 (7.7%)	
Gestational weight gain^j^				
Inadequate	145 (16.0%)			
Adequate	345 (38.0%)			
Excessive	418 (46.0%)			

p-values were obtained from comparison between GWG reporters and GWG non-reporters using t-tests for continuous variables and chi-squared tests for categorical variables.

^a^ Gestational Weight Gain

^b^ Non-reporters includes ten participants who reported their gestational weight gain but not their pre-pregnancy BMI and therefore could not be included in gestational weight gain analyses.

^c^ Other includes including Hispanic, Asian/Pacific Islander, multiracial, and other races

^d^ Poverty income ratio ≤1.85

^e^ Poverty income ratio >1.85 and <4

^f^ Poverty income ratio ≥4

^g^ Mother participated in Women, Infants, and Children (WIC) during pregnancy

^h^ Alternative Healthy Eating Index for Pregnancy

^i^ Body Mass Index. These body mass index ranges are generally categorized as underweight, normal weight, overweight, and obese

^j^ Gestational weight gain category was determined according to the 2009 Institute of Medicine’s pre-pregnancy body mass index-dependent gestational weight gain recommendations; adequate GWG ranges for each pre-pregnancy BMI group are 28–40 lb for underweight, 25–35 lb for normal weight, 15–25 lb for overweight, and 11–20 lb for obese

### Pre-pregnancy BMI

In both age- and multivariable-adjusted models, pre-pregnancy BMI was associated with total AHEI-P scores (**Table 2**). In the age-adjusted model, women with underweight (mean ± standard error (SE) = 63.2±1.3 points), normal weight (62.5±0.4 points) and overweight (60.6±0.6 points) scored similarly to each other; women with underweight and normal weight, scored significantly higher than women with obesity (59.1±0.6 points). In the multivariable-adjusted model, similar to the age-adjusted model, women with underweight (60.9±1.4 points) and normal weight (60.6±0.8 points) scored similarly; women with normal weight scored significantly higher than both women with overweight (58.9±0.7 points) and obesity (58.4±0.8 points). Differences between women with underweight and women with obesity were no longer significant. The interactions tested between pre-pregnancy BMI and poverty income and energy intake were non-significant.

**Table 2:** Age and multivariable adjusted Alternate Healthy Eating Index for Pregnancy total and component scores by pre-pregnancy BMI of participants in the Infant Feeding Practices Study II (n = 1268).

	Total AHEI-P Score^1^	Vegetables, servings/d	Whole fruit, servings/d	Whole grains, g/d	Sugar sweetened beverages,^2^ servings/d	Nuts and legumes, servings/d	Red and processed meat, servings/d	Trans fat, % of energy	Long chain (n = 3) fats,^3^ mg/d	PUFA,^4^ % of energy	Sodium, mg/d	Iron, mg/d	Folate, mg/d	Calcium, mg/d
**Age-adjusted model (n = 1268)**														
BMI<18.5^6^	63.2±1.3^a^	6.0±0.3	4.3±0.3^a^	2.9±0.2	1.5±0.4	2.7±0.3	4.3±0.4	4.7±0.2	2.9±0.3	6.3±0.2	4.2±0.4	6.9±0.2^a^	7.4±0.3^a^	9.0±0.2
BMI≥18.5–24.9^7^	62.5±0.4^a^	5.4±0.1	3.9±0.1^a^	2.9±0.1^a^	2.1±0.1	2.7±0.1	4.5±0.1	5.0±0.1^a^	2.5±0.1	6.5±0.1	5.0±0.1	6.4±0.1^a^	6.9±0.1^a,c^	8.6±0.1
BMI≥25.0–29.9 ^8^	60.6±0.6^a,b^	5.0±0.2	3.7±0.2	2.7±0.1	2.0±0.2	2.6±0.1	4.2±0.2	4.8±0.1	2.6±0.1	6.3±0.1	5.0±0.2	6.2±0.1^a^	6.6±0.1^b,c^	8.7±0.1
BMI≥30.0^9^	59.1±0.6^b^	5.3±0.2	3.3±0.1^b^	2.4±0.1^b^	2.2±0.2	2.5±0.1	3.9±0.2	4.6±0.1^b^	2.6±0.1	6.6±0.1	5.1±0.2	5.8±0.1^b^	6.3±0.1^b^	8.4±0.1
**Multivariable-adjusted model–adjusted for age, smoking, poverty income ratio, WIC participation**^5^**, race, and kcals (n = 1252)**														
BMI<18.5	60.9±1.4^a,c^	5.5±0.4	4.2±0.4	2.5±0.2	1.3±0.4	2.1±0.3	4.4±0.4	4.8±0.2	3.0±0.3	6.4±0.2	5.2±0.3	6.3±0.2^a^	6.9±0.2^a,b^	8.2±0.2
BMI≥18.5–24.9	60.6±0.8^a^	5.3±0.2	3.9±0.2	2.5±0.1^a^	1.7±0.2	2.3±0.2	4.2±0.2^a^	5.1±0.1^a^	2.7±0.2	6.6±0.1	5.2±0.1	6.3±0.1^a^	6.8±0.1^a^	8.2±0.1
BMI≥25.0–29.5	58.9±0.7^b,c^	5.0±0.2	3.8±0.2	2.3±0.1	1.6±0.3	2.2±0.2	3.9±0.2	4.9±0.1	2.8±0.2	6.4±0.2	5.3±0.2	6.1±0.1^a^	6.5±0.1^b,c^	8.2±0.1
BMI≥30.0	58.4±0.8^b,c^	5.3±0.2	3.6±0.2	2.1±0.1^b^	1.9±0.3	2.2±0.2	3.7±0.2^b^	4.7±0.1^b^	2.9±0.2	6.7±0.1	5.2±0.1	5.7±0.1^b^	6.3±0.1^c^	8.0±0.1

Scores with different superscripted letters indicate significant post hoc differences between groups (p<0.05). Higher scores indicate better adherence to dietary recommendations. Inverse scoring (where lower consumption equates to a higher score) was used for the following categories: sugar sweetened beverages (and fruit juice), red and processed meat, trans fat, and sodium.

^1^Component scores are out of 10 possible points, total scores are out of 130 possible points

^2^ Sugar sweetened beverages includes fruit juices

^3^ Long chain fats component consists of EPA and DHA

^4^ Polyunsaturated fatty acids

^5^Women, Infants, and Children participant during pregnancy.

^6^ Body mass index (BMI) < 18.5 is classified as underweight (n = 66)

^7^ BMI ≥ 18.5–24.9 is classified as normal weight (n = 596)

^8^ BMI ≥ 25.0–29.9 is classified as overweight (n = 285)

^9^ BMI ≥ 30 is classified as obese (n = 321).

Women with pre-pregnancy underweight, normal weight, and overweight scored higher than women with pre-pregnancy obesity (p<0.05) on numerous AHEI-P components in both age-and multivariable-adjusted analyses. In age- and multivariable-adjusted analyses, women with normal weight scored about half a point higher than women with obesity in whole fruit (age-adjusted only), whole grains, red and processed meat (multivariable-adjusted only), *trans* fat, iron, and folate components. Women with underweight scored over a half a point higher than women with obesity in age-adjusted comparisons of whole fruit and in age- and multivariable-adjusted comparisons of both iron and folate. Women with overweight scored about half a point higher than women with obesity on the iron component in both age- and multivariable adjusted analyses.

### Gestational weight gain

Gestational weight gain was associated with total AHEI-P scores in both age- and multivariable-adjusted models (**Table 3**), however, no significant post-hoc differences were detected between GWG groups. In both models, women with adequate GWG scored descriptively highest (mean±SE, age-adjusted = 62.2±0.6 points, multivariable-adjusted = 61.0±0.5 points), followed by excessive GWG (age-adjusted = 61.0±0.5 points, multivariable-adjusted = 59.7±0.8 points), and inadequate GWG scored lowest (age-adjusted = 60.0±0.9 points, multivariable-adjusted = 59.3±1.0 points). The interaction between GWG and race was insignificant but the interaction between GWG and pre-pregnancy BMI was significant (p = 0.04), therefore, GWG models were stratified by pre-pregnancy BMI group. In the stratified age-adjusted models (**Table 4**), GWG was associated with total AHEI-P scores in the underweight and normal weight (p = 0.01) models but not in the overweight (p = 0.13) and obese (p = 0.08) models; a single significant post-hoc difference was observed in the underweight model between inadequate (55.3±3.1 points) and excessive (66.8±2.4 points) GWG. After multivariable adjustment, GWG was associated with AHEI-P scores in all pre-pregnancy BMI groups (p<0.05), however, significant post-hoc differences in total scores were attenuated with further adjustment.

**Table 3:** Age and multivariable adjusted Alternate Healthy Eating Index for Pregnancy total and component scores by Gestational Weight Gain of participants in the IFPS II (n = 908).

	Total AHEI-P Score^1^	Vegetables, servings/d	Whole fruit, servings/d	Whole grains, g/d	Sugar sweetened beverages,^2^ servings/d	Nuts and legumes, servings/d	Red and processed meat, servings/d	Trans fat, % of energy	Long chain (n = 3) fats,^3^ mg/d	PUFA,^4^ % of energy	Sodium, mg/d	Iron, mg/d	Folate, mg/d	Calcium, mg/d
**Age-adjusted model (n = 908)**														
Inadequate^6^	60.0±0.9	5.1±0.2	3.3±0.2^a^	2.7±0.1	2.3±0.3	2.4±0.2	4.4±0.2	4.7±0.1	2.5±0.2	6.5±0.1	5.2±0.3	6.0±0.2	6.5±0.2	8.3±0.2
Adequate^7^	62.2±0.6	5.1±0.1	3.8±0.1	2.8±0.1	2.3±0.2	2.7±0.1	4.7±0.2	5.0±0.1	2.5±0.1	6.4±0.1	5.3±0.2	6.3±0.1	6.7±0.1	8.6±0.1
Excessive^8^	61.0±0.5	5.4±0.1	3.8±0.1^b^	2.7±0.1	1.8±0.2	2.7±0.1	4.3±0.1	4.8±0.1	2.6±0.1	6.6±0.1	4.9±0.2	6.2±0.1	6.6±0.1	8.7±0.1
**Multivariable-adjusted model–adjusted for age, smoking, parity, poverty income ratio, WIC participation**^5^**, and kcals (n = 890)**														
Inadequate	59.3±1.0	5.1±0.3	3.2±0.3	2.3±0.2	2.4±0.3	2.4±0.2	4.4±0.3	4.9±0.1	2.4±0.2	6.4±0.2	5.3±0.2	5.8±0.1	6.4±0.1	8.2±0.2
Adequate	61.0±0.8	5.0±0.2	3.6±0.2	2.5±0.1	2.3±0.3	2.7±0.2	4.7±0.2	5.1±0.1	2.4±0.2	6.4±0.1	5.4±0.1	6.1±0.1	6.6±0.1	8.4±0.1
Excessive	59.7±0.8	5.2±0.2	3.5±0.2	2.3±0.1	2.0±0.2	2.6±0.2	4.3±0.2	5.0±0.1	2.4±0.2	6.6±0.1	5.2±0.1	5.8±0.1	6.3±0.1	8.4±0.1

Scores with different superscripted letters indicate significant post hoc differences between groups (p<0.05). Higher scores indicate better adherence to dietary recommendations. Inverse scoring (where lower consumption equates to a higher score) was used for the following categories: sugar sweetened beverages (and fruit juice), red and processed meat, trans fat, and sodium.

^1^Component scores are out of 10 possible points, total scores are out of 130 possible points

^2^ Sugar sweetened beverages includes fruit juices

^3^ Long chain fats component consists of EPA and DHA

^4^ Polyunsaturated fatty acids

^5^Women, Infants, and Children participant during pregnancy.

^6^ Inadequate, n = 145

^7^ Adequate, n = 345

^8^ Excessive, n = 418.

Adequate GWG ranges for each pre-pregnancy BMI group are 28–40 lb for underweight, 25–35 lb for normal weight, 15–25 lb for overweight, and 11–20 lb for obese

**Table 4:** Complete case analyses of age and multivariable adjusted Alternate Healthy Eating Index for Pregnancy total and component scores by gestational weight gain stratified by pre-pregnancy BMI of participants in the IFPS II (n = 908).

	Total AHEI-P Score^1^	Vegetables, servings/d	Whole fruit, servings/d	Whole grains, g/d	Sugar sweetened beverages,^2^ servings/d	Nuts and legumes, servings/d	Red and processed meat, servings/d	Trans fat, % of energy	Long chain (n = 3) fats,^3^ mg/d	PUFA,^4^% of energy	Sodium, mg/d	Iron, mg/d	Folate, mg/d	Calcium, mg/d
**Age-adjusted model (n = 908)**														
**BMI < 18.5**^**6**^**(n = 44)**														
Inadequate (n = 9)	55.3±3.1^a^	4.6±0.9	3.4±1.0	2.1±0.5	0.6±0.8	1.7±0.5	4.6±1.1	4.9±0.5	2.4±0.8	6.0±0.6	5.9±0.9^a^	5.3±0.7^a^	6.2±0.7	7.6±0.6^a^
Adequate (n = 20)	61.7±2.1	4.8±0.6	4.2±0.6	3.2±0.4	0.9±0.5	2.2±0.3	5.0±0.7	4.8±0.4	2.5±0.5	5.9±0.4	4.8±0.6	7.0±0.5	7.3±0.5	9.2±0.4
Excessive (n = 15)	66.8±2.4^b^	6.5±0.7	4.7±0.7	3.9±0.4	1.5±0.6	3.2±0.4	4.0±0.8	4.9±0.4	3.3±0.6	5.9±0.4	3.0±0.7^b^	8.0±0.5^b^	8.4±0.6	9.5±0.4^b^
**BMI ≥18.5–24.9 (n = 431)**														
Inadequate (n = 65)	61.6±1.3	5.2±0.3	3.7±0.3	2.9±0.2	1.8±0.4	2.6±0.3	4.5±0.4	4.8±0.2	2.6±0.3	6.6±0.2	4.9±0.4	6.5±0.2	6.9±0.2	8.7±0.2
Adequate (n = 214)	63.6±0.7	5.2±0.2	4.1±0.2	3.0±0.1	2.3±0.2	2.7±0.2	4.9±0.2	5.2±0.1	2.5±0.2	6.4±0.1	5.3±0.2	6.4±0.1	6.9±0.1	8.5±0.1
Excessive (n = 152)	61.7±0.8	5.4±0.2	3.8±0.2	2.7±0.1	1.8±0.3	2.7±0.2	4.5±0.2	4.9±0.1	2.6±0.2	6.8±0.1	5.1±0.3	6.2±0.2	6.6±0.2	8.5±0.2
**BMI ≥25.0–29.9 (n = 209)**														
Inadequate (n = 13)	64.5±2.9	5.4±0.7	3.2±0.7	3.2±0.5	2.9±0.9	4.2±0.6	2.8±0.8^a^	4.4±0.4	3.4±0.6	7.2±0.5	4.2±0.9	7.1±0.5	7.5±0.6	8.9±0.5
Adequate (n = 65)	60.3±1.3	4.8±0.3	3.1±0.3	2.6±0.2	2.3±0.4	2.6±0.3	5.1±0.3^b^	4.7±0.2	2.4±0.2	6.3±0.2	5.4±0.4	6.1±0.2	6.3±0.2	8.5±0.2
Excessive (n = 131)	60.2±0.9	5.0±0.2	3.9±0.2	2.7±0.1	1.8±0.3	2.7±0.2	4.0±0.2^a^	4.9±0.1	2.4±0.2	6.3±0.1	4.9±0.3	6.2±0.2	6.6±0.2	8.8±0.2
**BMI ≥30.0(n = 224)**														
Inadequate (n = 58)	57.9±1.4	5.0±0.4	2.8±0.3	2.4±0.2	3.0±0.4	1.8±0.3^a^	4.6±0.4^a^	4.6±0.2	2.3±0.3	6.3±0.2	5.8±0.4	5.4±0.2	6.0±0.3	7.9±0.3^a^
Adequate (n = 46)	57.9±1.6	5.0±0.4	3.3±0.4	2.0±0.2	2.4±0.5	2.9±0.3^b^	2.8±0.4^b^	4.5±0.2	2.7±0.3	6.7±0.2	4.8±0.5	5.8±0.3	6.4±0.3	8.6±0.3
Excessive (n = 120)	60.3±1.0	5.6±0.3	3.7±0.2	2.5±0.1	1.9±0.3	2.6±0.2	4.3±0.3^a^	4.6±0.1	2.6±0.2	6.8±0.2	4.9±0.3	5.9±0.2	6.3±0.2	8.7±0.2^b^
**Multivariable-adjusted model–adjusted for age, smoking, parity, poverty income ratio, WIC participation**^5^**, and kcals (n = 890)**														
**BMI < 18.5**														
Inadequate	54.4±3.9	4.2±1.3	4.5±1.1	1.9±0.7	0.0±1.0	1.2±0.7	5.3±1.3	5.0±0.7	1.9±1.2	5.7±0.8	6.2±0.9	5.8±0.7	6.5±0.8	7.1±0.7
Adequate	59.7±3.6	4.2±1.2	4.9±1.0	2.7±0.6	0.1±0.9	1.6±0.7	6.2±1.2	4.5±0.7	1.8±1.1	6.0±0.7	6.1±0.8	6.8±0.7	6.8±0.7	8.0±0.7
Excessive	63.5±3.5	5.5±1.2	5.0±1.0	3.0±0.6	1.7±0.9	2.6±0.7	5.2±1.2	4.7±0.7	2.8±1.1	6.3±0.7	5.6±0.8	6.6±0.7	6.7±0.7	8.0±0.7
**BMI ≥18.5–24.9**														
Inadequate	59.2±1.5	4.9±0.4	3.4±0.4	2.4±0.2	1.9±0.5	2.4±0.3	4.5±0.4	5.0±0.2	2.3±0.3	6.3±0.3	5.3±0.3	5.9±0.2	6.5±0.2	8.3±0.2
Adequate	61.0±1.2	5.0±0.3	3.7±0.3	2.5±0.2	2.2±0.4	2.5±0.3	4.8±0.3	5.4±0.2	2.2±0.2	6.2±0.2	5.6±0.2	6.0±0.2	6.6±0.2	8.2±0.2
Excessive	59.3±1.2	5.2±0.3	3.4±0.3	2.3±0.2	1.8±0.4	2.6±0.3	4.3±0.3	5.2±0.2	2.4±0.3	6.6±0.2	5.3±0.2	5.8±0.2	6.4±0.2	8.2±0.2
**BMI ≥25.0–29.9**														
Inadequate	62.3±2.9	4.9±0.7	2.9±0.7	2.6±0.5	2.7±1.0	4.0±0.7	3.3±0.8	4.7±0.5	3.5±0.6	7.1±0.5	5.0±0.5	6.4±0.4	6.9±0.4	8.5±0.5
Adequate	60.7±1.6	4.8±0.4	3.2±0.4	2.3±0.3	2.5±0.5	3.0±0.4	4.9±0.4^a^	4.9±0.3	2.7±0.3	6.5±0.3	5.4±0.3	5.9±0.2	6.2±0.2	8.5±0.3
Excessive	59.4±1.5	4.6±0.4	3.7±0.4	2.2±0.3	2.0±0.5	2.9±0.4	3.9±0.4^b^	5.1±0.3	2.6±0.3	6.5±0.3	5.4±0.3	5.7±0.2	6.2±0.2	8.5±0.3
**BMI ≥30.0**														
Inadequate	59.2±1.8	5.5±0.4	2.8±0.4	2.4±0.2	3.5±0.6	2.0±0.4	4.6±0.5^a^	4.7±0.3	2.2±0.4	6.3±0.3	5.1±0.3	5.6±0.2	6.2±0.2	8.2±0.3
Adequate	57.8±2.0	5.2±0.5	3.1±0.5	1.9±0.2	2.9±0.7	2.9±0.4	2.9±0.5^b^	4.6±0.3	2.5±0.4	6.7±0.3	4.6±0.3	5.7±0.2	6.2±0.2	8.5±0.3
Excessive	60.6±1.5	5.9±0.4	3.5±0.3	2.3±0.2	2.4±0.5	2.6±0.3	4.3±0.4^a^	4.8±0.2	2.5±0.3	6.8±0.2	4.8±0.3	5.8±0.2	6.2±0.2	8.8±0.2

Scores with different superscripted letters indicate significant post-hoc differences between groups (p<0.05). Comparisons were tested within body mass index (BMI) groups only and not across BMI groups. Higher scores indicate better adherence to dietary recommendations. Inverse scoring (where lower consumption equates to a higher score) was used for the following categories: sugar sweetened beverages (and fruit juice), red and processed meat, trans fat, and sodium.

^1^Component scores are out of 10 possible points, total scores are out of 130 possible points

^2^ Sugar sweetened beverages includes fruit juices

^3^ Long chain fats component consists of EPA and DHA

^4^ Polyunsaturated fatty acids

^5^Women, Infants, and Children participant during pregnancy.

^6^ BMI < 18.5 is classified as underweight, BMI ≥ 18.5–24.9 is classified as normal weight, BMI ≥ 25.0–29.9 is classified as overweight, BMI ≥ 30 is classified as obese.

In analyses of women with pre-pregnancy underweight, overweight, and obesity, significant (p<0.05) age-adjusted post-hoc differences between GWG groups were observed in several components (including sodium, iron, calcium, nuts and legumes, and red and processed meat), however, most differences were attenuated and no longer significant (p>0.05) in multivariable-adjusted comparisons. In age-adjusted analysis of women with pre-pregnancy underweight, excessive GWG scored 1.9–2.7 points higher than inadequate GWG in calcium and iron and 2.9 points lower than inadequate GWG in sodium; these comparisons were no longer significant after further adjustment. In analysis of women with pre-pregnancy overweight, red and processed meat component scores were higher in adequate GWG compared to inadequate GWG in age-adjusted models and higher in adequate GWG compared to excessive GWG in both age- and multivariable-adjusted models. In age-adjusted models examining women with pre-pregnancy obesity, adequate GWG scored over a point higher than inadequate GWG in nuts and legumes and excessive GWG scored about a point higher than inadequate GWG in calcium. In age- and multivariable-adjusted analyses of women with pre-pregnancy obesity, red and processed meat scores for women with adequate GWG were 1.4–1.8 points lower than both inadequate and excessive GWG. Results from the multiple imputation analyses were consistent with the complete case analyses (**Table 5**).

**Table 5:** Multiple imputation analyses of age and multivariable adjusted Alternate Healthy Eating Index for Pregnancy total and component scores by gestational weight gain stratified by pre-pregnancy BMI of participants in the IFPS II.

	Total AHEI-P Score^1^	Vegetables, servings/d	Whole fruit, servings/d	Whole grains, g/d	Sugar sweetened beverages,^2^ servings/d	Nuts and legumes, servings/d	Red and processed meat, servings/d	Trans fat, % of energy	Long chain (n = 3) fats,^3^ mg/d	PUFA,^4^% of energy	Sodium, mg/d	Iron, mg/d	Folate, mg/d	Calcium, mg/d
**Age-adjusted model**														
**BMI < 18.5**^**6**^														
Inadequate	57.3±2.8^a^	5.2±0.9	3.6±0.8	2.1±0.5	0.8±0.7	2.0±0.6	4.3±1.0	4.6±0.5	2.7±0.9	6.3±0.5	5.2±0.9	5.9±0.7^a^	6.7±0.7	8.1±0.5^a^
Adequate	61.7±2.0	5.3±0.6	4.1±0.6	2.9±0.4	1.0±0.5	2.3±0.4	4.6±0.7	4.7±0.3	2.6±0.6	6.1±0.3	4.5±0.6	7.0±0.5	7.3±0.5	9.2±0.3
Excessive	65.6±2.2^b^	6.7±0.6	4.4±0.6	3.4±0.4	1.3±0.5	3.3±0.5	3.8±0.7	4.8±0.3	3.2±0.6	6.2±0.3	3.1±0.6	7.7±0.5^b^	8.2±0.5	9.4±0.3^b^
**BMI ≥18.5–24.9**														
Inadequate	62.1±1.2	5.5±0.3	3.7±0.3	3.0±0.2	1.8±0.3	2.7±0.3	4.3±0.3	4.8±0.2	2.6±0.3	6.5±0.2	4.6±0.3	6.7±0.2	7.1±0.2	8.8±0.2
Adequate	63.2±0.7	5.2±0.2	4.1±0.2	3.0±0.1	2.3±0.2	2.7±0.2	4.8±0.2	5.1±0.1	2.5±0.1	6.4±0.1	5.3±0.2	6.5±0.1	6.9±0.1	8.6±0.1
Excessive	61.8±0.8	5.5±0.2	3.7±0.2	2.7±0.1	2.0±0.2	2.7±0.2	4.3±0.2	4.9±0.1	2.6±0.2	6.7±0.1	4.8±0.2	6.3±0.1	6.8±0.1	8.6±0.1
**BMI ≥25.0–29.9**														
Inadequate	62.2±2.7	5.2±0.7	3.1±0.7	2.8±0.4	2.7±0.8	3.3±0.6	3.4±0.8	4.6±0.4	3.2±0.6	6.9±0.5	4.9±0.9	6.6±0.5	6.9±0.6	8.6±0.5
Adequate	60.7±1.3	4.9±0.3	3.3±0.3	2.6±0.2	2.3±0.4	2.6±0.3	5.0±0.3^a^	4.7±0.2	2.5±0.3	6.3±0.2	5.4±0.4	6.1±0.2	6.4±0.3	8.5±0.2
Excessive	60.8±0.9	5.1±0.2	4.0±0.2	2.7±0.1	1.9±0.3	2.6±0.2	4.0±0.2^b^	4.9±0.1	2.6±0.2	6.3±0.1	4.9±0.3	6.3±0.2	6.7±0.2	8.7±0.2
**BMI ≥30.0**														
Inadequate	58.2±1.4	5.1±0.4	2.9±0.3	2.5±0.2	2.9±0.5	2.0±0.3^a^	4.3±0.4^a^	4.6±0.2	2.4±0.3	6.4±0.2	5.5±0.4	5.6±0.2	6.1±0.3	8.1±0.3
Adequate	58.5±1.5	5.3±0.4	3.3±0.4	2.2±0.2	2.3±0.5	2.9±0.3^b^	3.0±0.4^b^	4.5±0.2	2.7±0.3	6.8±0.3	4.8±0.4	5.8±0.3	6.4±0.3	8.4±0.3
Excessive	60.4±0.9	5.6±0.2	3.6±0.2	2.5±0.1	1.9±0.3	2.7±0.2	4.1±0.2^a^	4.7±0.1	2.7±0.2	6.7±0.1	4.9±0.3	5.9±0.2	6.4±0.2	8.6±0.2
**Multi-variable adjusted model–adjusted for age, smoking, parity, poverty income ratio, WIC participation**^5^**, and kcals**														
**BMI < 18.5**														
Inadequate	59.8±3.6	5.8±1.1	4.9±1.0	2.1±0.6	0.0±0.9	1.7±0.7	5.0±1.1	4.7±0.5	2.9±1.0	6.2±0.6	4.8±0.8	6.5±0.7	7.4±0.7	8.0±0.6
Adequate	63.2±2.8	5.6±0.8	5.2±0.7	2.6±0.5	0.3±0.7	2.0±0.6	5.5±0.9	4.5±0.4	2.8±0.8	6.3±0.5	4.8±0.6	7.2±0.5	7.6±0.6	8.8±0.4
Excessive	66.2±2.7	6.7±0.8	5.1±0.7	3.0±0.5	1.3±0.7	3.0±0.5	4.9±0.8	4.5±0.4	3.2±0.8	6.6±0.5	4.0±0.6	7.3±0.5	7.8±0.5	8.7±0.4
**BMI ≥18.5–24.9**														
Inadequate	60.4±1.5	5.3±0.3	3.5±0.4	2.6±0.2	1.8±0.4	2.5±0.3	4.4±0.3	5.2±0.2	2.3±0.3	6.3±0.2	5.1±0.2	6.2±0.2	6.8±0.2	8.5±0.2
Adequate	61.7±1.1	5.2±0.3	3.9±0.3	2.6±0.2	2.1±0.3	2.5±0.2	4.7±0.3	5.5±0.1	2.2±0.2	6.2±0.2	5.3±0.2	6.3±0.2	6.9±0.1	8.4±0.2
Excessive	60.1±1.1	5.4±0.3	3.5±0.3	2.4±0.2	1.9±0.3	2.5±0.2	4.3±0.3	5.2±0.1	2.3±0.2	6.4±0.2	5.1±0.2	6.1±0.2	6.6±0.1	8.3±0.2
**BMI ≥25.0–29.9**														
Inadequate	62.1±2.5	5.1±0.7	3.1±0.6	2.5±0.4	2.6±0.9	3.5±0.6	3.5±0.8	4.8±0.4	3.2±0.6	6.9±0.5	5.1±0.5	6.3±0.4	6.8±0.4	8.7±0.4
Adequate	60.7±1.4	4.9±0.4	3.2±0.3	2.4±0.2	2.2±0.5	2.8±0.3	4.9±0.4^a^	4.9±0.2	2.6±0.3	6.3±0.3	5.3±0.3	6.0±0.2	6.4±0.2	8.8±0.2
Excessive	59.6±1.3	4.8±0.3	3.7±0.3	2.3±0.2	1.9±0.4	2.6±0.3	4.0±0.4^b^	5.0±0.2	2.6±0.3	6.4±0.2	5.3±0.2	5.9±0.2	6.4±0.2	8.8±0.2
**BMI ≥30.0**														
Inadequate	59.3±1.6	5.5±0.4	3.0±0.4	2.6±0.2	3.3±0.5	2.1±0.3	4.3±0.4^a^	4.7±0.2	2.2±0.3	6.4±0.3	5.1±0.3	5.8±0.2	6.3±0.2	8.1±0.3
Adequate	58.9±1.7	5.4±0.4	3.3±0.4	2.2±0.2	2.8±0.6	2.9±0.4	3.1±0.4^b^	4.7±0.3	2.6±0.4	6.8±0.3	4.7±0.3	5.9±0.2	6.4±0.2	8.3±0.3
Excessive	60.5±1.1	5.7±0.3	3.5±0.3	2.5±0.2	2.4±0.4	2.6±0.3	4.1±0.3^a^	4.8±0.2	2.5±0.3	6.8±0.2	4.9±0.2	5.9±0.1	6.4±0.2	8.5±0.2

Scores with different superscripted letters indicate significant post-hoc differences between groups (p<0.05). Comparisons were tested within body mass index (BMI) groups only and not across BMI groups. Higher scores indicate better adherence to dietary recommendations. Inverse scoring (where lower consumption equates to a higher score) was used for the following categories: sugar sweetened beverages (and fruit juice), red and processed meat, trans fat, and sodium.

^1^Component scores are out of 10 possible points, total scores are out of 130 possible points

^2^ Sugar sweetened beverages includes fruit juices

^3^ Long chain fats component consists of EPA and DHA

^4^ Polyunsaturated fatty acids

^5^Women, Infants, and Children participant during pregnancy.

^6^ BMI < 18.5 is classified as underweight, BMI ≥ 18.5–24.9 is classified as normal weight, BMI ≥ 25.0–29.9 is classified as overweight, BMI ≥ 30 is classified as obese.

## Discussion

This study examined the relationships between pre-pregnancy BMI, GWG, and prenatal diet quality within participants in the Infant Feeding Practices Study II, a national United States convenience sample. Consistent with previous studies [15,20,38,39], we observed low total prenatal diet quality in the overall sample (mean±SE = 61.2±10.8 points out of 130) though some groups were disproportionately affected such as women with pre-pregnancy overweight and obesity who scored 1.9–2.5 points lower than women with lower pre-pregnancy BMIs. In examining GWG, women with adequate GWG descriptively scored 1.3–1.7 points higher than women with inadequate and excessive GWG, however, the relationship between GWG adherence and prenatal diet quality was dependent on pre-pregnancy BMI as indicated by a significant GWG and pre-pregnancy BMI interaction. While diet quality changes of 5% are generally considered clinically-meaningful [40], the smaller differences in diet quality (of 1.3–2.5 points or 1–1.9%) present between BMI and GWG groups in this observational study suggest that pre-pregnancy BMI and GWG are correlated with diet quality. The associations observed suggest that interventions specifically targeting high-risk pre-pregnancy BMI and/or GWG groups may potentially lead to meaningful improvements in diet quality.

Similar to other studies, we found women with pre-pregnancy overweight and obesity were at increased risk of poor prenatal diet quality [15,19,20]. Component score analyses indicated that pre-pregnancy normal weight compared to pre-pregnancy obesity was associated with more desirable scores in 2 of 4 moderation (red and processed meat and *trans* fat) and 3 of 9 adequacy (whole grains, iron, and folate) AHEI-P components. Using a previous version of the AHEI-P in a regional sample of pregnant women, Rifas-Shiman et al. similarly observed a significant inverse relationship between pre-pregnancy BMI and component scores in 1 of 1 moderation (trans fat) and 5 of 8 adequacy components (fruit, red to white meat ratio (red and processed meat in the updated AHEI-P), fiber (whole grains in the updated AHEI-P), calcium, and folate) [20]. Together, these component score findings suggest that BMI disparities in prenatal diet quality may be influenced by the consumption of holistically healthier diets (i.e., containing more micronutrient-rich, healthful foods and less highly processed, unhealthful foods) in women with lower pre-pregnancy BMIs.

At any pre-pregnancy BMI, adhering to GWG and dietary guidelines can reduce risks of adverse outcomes [2,27,41,42], however, we found the relationship between GWG and diet quality to be differently associated across pre-pregnancy BMI groups. Though not significant, we observed unique descriptive diet quality differences across GWG groups in pre-pregnancy BMI-stratified models. We hypothesized that adequate GWG would be associated with higher diet quality, yet this finding was only observed in women with pre-pregnancy normal weight where adequate GWG scored descriptively highest (by 1.7–1.8 points) on the AHEI-P. In women with underweight and obesity, diet quality was descriptively highest (by 2.8–3.8 points) in the excessive GWG group. The impacts of excessive GWG on pregnancy outcomes in women with underweight is controversial; while some studies advocate for unrestricted GWG to mitigate risk of low birth weight [43–45], various studies informing the Institute of Medicine’s recommendations emphasize that excessive GWG in underweight women increases the risk of macrosomia [30,46,47]. While this study examined the association between GWG and diet quality, further research should examine whether high prenatal diet quality can mitigate the risk of adverse pregnancy outcomes associated with non-optimal GWG. In women with pre-pregnancy obesity, high diet quality in the excessive GWG group may be related to the narrow GWG recommendation for this BMI group (11–20 lb (5.0–9.1 kg)), making adherence challenging. The Institute of Medicine recognizes that the GWG recommendations will commonly be exceeded for women with pre-pregnancy overweight and obesity and that interventions will be needed to increase adherence [30]. Though we observed descriptively higher diet quality among women with overweight who experienced inadequate GWG, our findings support the need for interventions targeting women with pre-pregnancy overweight and obesity who had the lowest diet quality in our BMI analysis. However, further research should also consider the relative importance of and potential interactions between prenatal diet quality, GWG, pre-pregnancy BMI and their combined influence on health outcomes.

The present study contributes to the current understanding of the relationship between GWG and prenatal diet quality, which, to our knowledge, has only been examined in two previous studies. In an analysis of nationally representative data, Shin et al. did not find a significant association between prenatal HEI-2005 scores and GWG, however, GWG was assessed at various points in pregnancy rather than cumulatively at the end of pregnancy [48]. Similarly, in a clinical sample of 41 women, Grandy et al. did not observe an association between 3^rd^ trimester diet quality and GWG.[49] Though we examined total GWG in our relatively large sample, about 31% of participants were missing GWG data and since substantial missing data can lead to bias in complete case analyses [50], sensitivity analyses using multiply imputed data were completed. Findings for both the complete case and multiply imputed analyses indicated significant associations between GWG adherence (stratified by BMI group) and AHEI-P scores. Despite a lack of significant post-hoc differences observed in our complete case and multiply imputed GWG models, the significance observed in the overall models and the different descriptive patterns observed across pre-pregnancy BMI groups suggests that GWG may be an important consideration in monitoring prenatal diet quality.

Women are encouraged to conceive within the normal weight BMI range to reduce risk of adverse outcomes related to pregnancy, birth, and long-term child health [30,51,52]. However, in the US, it is estimated that approximately two thirds of women of childbearing age are overweight and about one third of these women are further categorized as obese [30]. While critical to improving long-term health outcomes, it is currently unclear how to best reduce the prevalence of pre-pregnancy overweight and obesity as very few studies have examined pre-conceptional weight loss interventions. A 2013 systematic review on pre-conceptual weight loss interventions only included 2 studies and the authors speculated that practical challenges involved with recruiting women prior to pregnancy contributed to the paucity of research [53]. Targeting excessive GWG, rather than pre-pregnancy overweight and obesity, may be more feasible for engaging the target population. A 2017 meta-analysis of individual patient data from 33 randomized control trials indicated that women participating in prenatal diet and exercise interventions gained approximately 1.54 lb (0.7 kg) less than women in control groups[54]. Although the interventions targeted excess energy intake and/or low physical activity, known risk factors for excess GWG [55], prenatal diet quality was rarely targeted or assessed in the included intervention studies. Therefore, further research is needed to characterize the dynamic and potentially interactive relationship between GWG and prenatal diet quality and their influence on health outcomes.

While the present study examined how pre-pregnancy BMI and GWG were related to diet quality, it’s possible that targeting prenatal diet quality may be a more prudent population health target than exclusively focusing on reducing pre-pregnancy overweight/obesity and promoting adequate GWG. Improving diet quality may help to promote adequate GWG by reducing energy intake [56] and independent of pre-pregnancy BMI, prenatal diet quality has been associated with a variety of improved health outcomes such as lower fetal adiposity, and lower risk of preeclampsia and some congenital malformations [2,41,42]. Furthermore, interventions targeting prenatal diet quality may promote adequate GWG and might also offer benefits in terms of reducing postpartum weight retention and encouraging/modeling healthy eating habits to children. While further research is needed to determine the most impactful and feasible prenatal public health targets, clinicians can encourage high quality diets, physical activity, and adherence to GWG guidelines in women at all pre-pregnancy BMIs.

This analysis is not without limitations. There were substantial missing data for GWG in the sample, however, sensitivity analyses with multiply imputed data were completed and findings were similar between the complete case and multiply imputed analyses. The guidelines used to assess GWG adherence were published in 2009 after the IFPS II data was collected. With the exception of the range for women with pre-pregnancy obesity which changed from ≥15 lb (6.8 kg). in 1990 to 11–20 lb (5.0–9.1 kg) in 2009, recommended GWG ranges in the 2009 update were identical to the previous 1990 guidelines though it should be noted that the previous guidelines used different BMI classifications (in 1990 guidelines, underweight<19.8, normal weight = 19.8–26.0, overweight = 26.1–29.0, obese>29.0) [30]. While the IFPS II is an older dataset comprised of self-reported measures, this larger dataset includes detailed dietary, health, and demographic information in a national convenience sample of pregnant women, though some subgroups (e.g. underweight) may have been too small to detect differences. A variety of relevant variables were controlled for in analyses; however, some important social and lifestyle factors, such as such as sleep quality [57], stress, depression, social support [58,59], and built environment [60], were not assessed in IFPS II and therefore, were not controlled for in analyses.

This analysis also has several strengths. To our knowledge, this is the first study to date to examine the relationship between total GWG and prenatal diet quality, providing critical insight into this understudied relationship. The IFPS II dataset provides detailed dietary, health, and demographic information, which allowed us to adjust for various influential covariates. The large sample size and national recruitment of women in IFPS II are also strengths of this analysis.

## Conclusions

Our findings show that the combined effects of pre-pregnancy BMI and GWG are important considerations when examining predictors of prenatal diet quality. Though further research is needed to fully understand the relationship between pre-pregnancy BMI, GWG, and prenatal diet quality, this study identified women with pre-pregnancy overweight and obesity as higher-risk groups for poor prenatal diet quality that should be targeted in future interventions. Our results support encouraging women to enter pregnancy at a normal weight BMI due to associations with higher diet quality, however, achieving adequate GWG and consuming a high-quality prenatal diet should be emphasized at any pre-pregnancy BMI.

## Supporting information

S1 Dataset IFPS II dataset. (CSV)
